# Effectiveness of early versus delayed rehabilitation following total shoulder replacement: A systematic review

**DOI:** 10.1177/02692155211044137

**Published:** 2021-11-01

**Authors:** Maria Moffatt, Gareth Whelan, Peter Gill, Bruno Mazuquin, Peter Edwards, Chris Peach, Ronnie Davies, Marie Morgan, Chris Littlewood

**Affiliations:** 1 5289 Manchester Metropolitan University, Manchester, UK; 2 8749 York Teaching Hospitals NHS Foundation Trust, York, UK; 3 523611 Northern Care Alliance NHS Group, Salford, UK; 4 5289 Manchester Metropolitan University, Manchester, UK; 5 1649 Curtin University, Perth, Australia; 6 98564 Manchester University Foundation Trust, Manchester, UK; 7 Manchester University Foundation Trust, Manchester, UK; 8 2102 University Hospitals of Derby and Burton NHS, Derby, UK; 9 5289 Manchester Metropolitan University, Manchester, UK

**Keywords:** Arthroplasty, shoulder, glenohumeral, replacement, rehabilitation, systematic review

## Abstract

**Objective:**

To investigate the effectiveness of early versus delayed rehabilitation following total shoulder replacement.

**Design:**

Intervention systematic review with narrative synthesis.

**Literature search:**

MEDLINE, EMBASE, CINAHL, Scopus and the Cochrane Library were searched from inception to the 29^th^ of July 2021.

**Study selection criteria:**

Randomised controlled trials comparing early versus delayed rehabilitation following primary anatomic, primary reverse, or revision total shoulder replacement.

**Data synthesis:**

A revised Cochrane risk of bias assessment tool for randomised controlled trials was used, as well as the Grading of Recommendations Assessment, Development and Evaluation approach to evaluate the quality of evidence. A narrative synthesis was undertaken.

**Results:**

Three eligible randomised controlled trials (*n* = 230) were included. There was very low-quality evidence of no statistically significant difference (*P* > 0.05) in pain, shoulder function, health-related quality of life or lesser tuberosity osteotomy healing at 12 months between early or delayed rehabilitation. There was conflicting and very low-quality evidence of a difference between the effect of early and delayed rehabilitation on shoulder range of movement. There was limited, very low-quality evidence of statistically significantly improved pain and function (*P* < 0.05) in the early post-operative period with early rehabilitation following anatomic total shoulder replacement.

**Conclusions:**

No differences were seen in patient-reported or clinician-reported outcomes at 12 months post-surgery between early and delayed rehabilitation following total shoulder replacement. There is very low-quality evidence that early rehabilitation may improve shoulder pain and function in the early post-operative phase following anatomic total shoulder replacement.

## Introduction

Total shoulder replacement is a treatment option for individuals experiencing severe pain and functional restriction of their shoulder. Two of the main indications for total shoulder replacement are osteoarthritis and cuff tear arthropathy.^1^ Between 1998 and 2017, over 58,000 elective shoulder replacements were undertaken in the United Kingdom (UK), and this number is rising annually.^2^ A similar trend has been reported in the United States (US),^3^ resulting in significant annual costs to both the UK National Health Service (NHS)^4^ and the US healthcare system.^5^

Following total shoulder replacement, a programme of rehabilitation is important to help patients achieve the best clinical and quality of life outcomes.^6,7^ However, the optimal approach to post-operative rehabilitation is unknown.^8^ Variation is observed across the UK NHS^9^ and few institutions adapt their rehabilitation protocol based on prosthesis type^9^ despite the common assertion that different approaches are necessary.^8^ One key uncertainty regarding rehabilitation relates to the best time to begin mobilisation of the shoulder after surgery. This has recently been identified as a research priority by the UK National Institute for Health and Care Excellence.^10^

A previous systematic review highlighted the paucity of high-quality evidence available relating to rehabilitation following total shoulder replacement.^11^ Only one randomised controlled trial was eligible for inclusion, which suggested that early rehabilitation might lead to a more rapid return of function following total shoulder replacement.^12^ Since this previous systematic review,^11^ further relevant randomised controlled trials have been published. The aim of this review was therefore to build on the previous systematic review and examine evidence from randomised controlled trials to investigate the effectiveness of early versus delayed rehabilitation on pain, function, and tissue healing in adults who have undergone primary anatomic, primary reverse, or revision total shoulder replacement.

## Methods

This systematic review was prospectively registered on PROSPERO (ID number: CRD42020208472 available from: https://www.crd.york.ac.uk/prospero/display_record.php?RecordID=208472) and reported according to the PRISMA statement.^13^

Included randomised controlled trials were selected according to the following eligibility criteria: Adults undergoing formal post-surgical rehabilitation following either elective primary anatomic or reverse total shoulder replacement, or elective revision total shoulder replacement; intervention involving early rehabilitation after total shoulder replacement (defined by the randomised controlled trial authors); comparator involving delayed rehabilitation after total shoulder replacement (defined by the randomised controlled trial authors); and outcomes consisting of any patient reported outcomes related to pain, disability, or function, and/or any objective measurement of range of movement, strength or tissue healing reported in the post-operative period.

A comprehensive literature search was undertaken via key databases: (MEDLINE, EMBASE, CINAHL, Scopus and the Cochrane Library) from inception until the 29th of July 2021 to identify relevant randomised controlled trials. Reference lists of potentially eligible studies were hand-searched. The grey literature was searched via OpenGrey and ClinicalTrials.gov. The search was limited to papers published in English. An example of the MeSH terms and keywords used for the searches are shown in Table 1. Full details of the search strategy are included in supplementary file 1**.**

**Table 1.** Mesh terms and keywords used for the MEDLINE database search.

Shoulder arthroplast * OR shoulder replacement* OR Glenohumeral arthroplasty* OR glenohumeral replacement* [Title/Abstract/Keywords]^a^ MESH Terms: Shoulder (Exploded), Shoulder joint (Exploded), Shoulder, arthroplasty, replacement (Exploded)
AND
Exercise* OR Rehab* OR Physiother* OR Physical ther* OR Telerehabilitation OR e-rehabilitation or m-rehabilitation [Title/Abstract/Keywords]^a^ MESH Terms: Physical therapy (Exploded), Exercise (Exploded)

a In each online database, advanced search options were selected to ensure MESH terms and keywords were searched for in article title, abstract and keywords.

All retrieved articles were imported into EndNote Online, and duplicates removed. Following this, the studies were uploaded to rayyan.qcri.org to enable independent screening of the titles and abstracts by two reviewers (MM and CL). Full texts of potentially eligible studies were independently reviewed by two reviewers (MM and CL) to determine inclusion. Disagreements were resolved through discussion.

Risk of bias assessment was undertaken by two independent reviewers (MM and GW) using the revised Cochrane risk-of-bias tool for randomised controlled trials (ROB-2). The risk of bias of all relevant outcomes was assessed individually.^14^ The ROB-2 includes five domains: (1) the randomisation process, (2) deviations from the intended intervention, (3) missing outcome data, 4) measurement of the outcome, and (5) selection of the reported result. Each domain was classified as ‘low risk’, ‘some concerns’ or ‘high risk’.^14^ Disagreements were resolved through discussion.

The Grading of Recommendations Assessment, Development and Evaluation (GRADE) system was used to rate the quality of evidence for each outcome. The initial GRADE assessment was undertaken by the first author (MM). This was reviewed and verified by a second author (CL). The quality of the evidence was downgraded when design limitations, indirectness of evidence, unexplained heterogeneity or inconsistency of results, imprecision of results, or publication bias were observed.^15^ Publication bias was not formally assessed using a funnel plot as no meta-analyses were possible.^16^

Data were extracted by the first author (MM) and entered into a pre-approved form in Microsoft Excel. This was then verified by a second author (PG). If the data provided in the published paper were deemed insufficient to facilitate statistical analysis, corresponding authors were contacted via email to request additional information. If no response was received after two weeks, a reminder email was sent. If no subsequent response was received, no further contact was made.

Where available, descriptive statistics were used to summarise variables extracted in the review. Included RCTs and responses from authors were reviewed to determine whether the data available and the level of clinical and methodological heterogeneity enabled a formal meta-analysis. If a meta-analysis was deemed inappropriate, a narrative synthesis would be undertaken.

## Results

**Figure 1.**
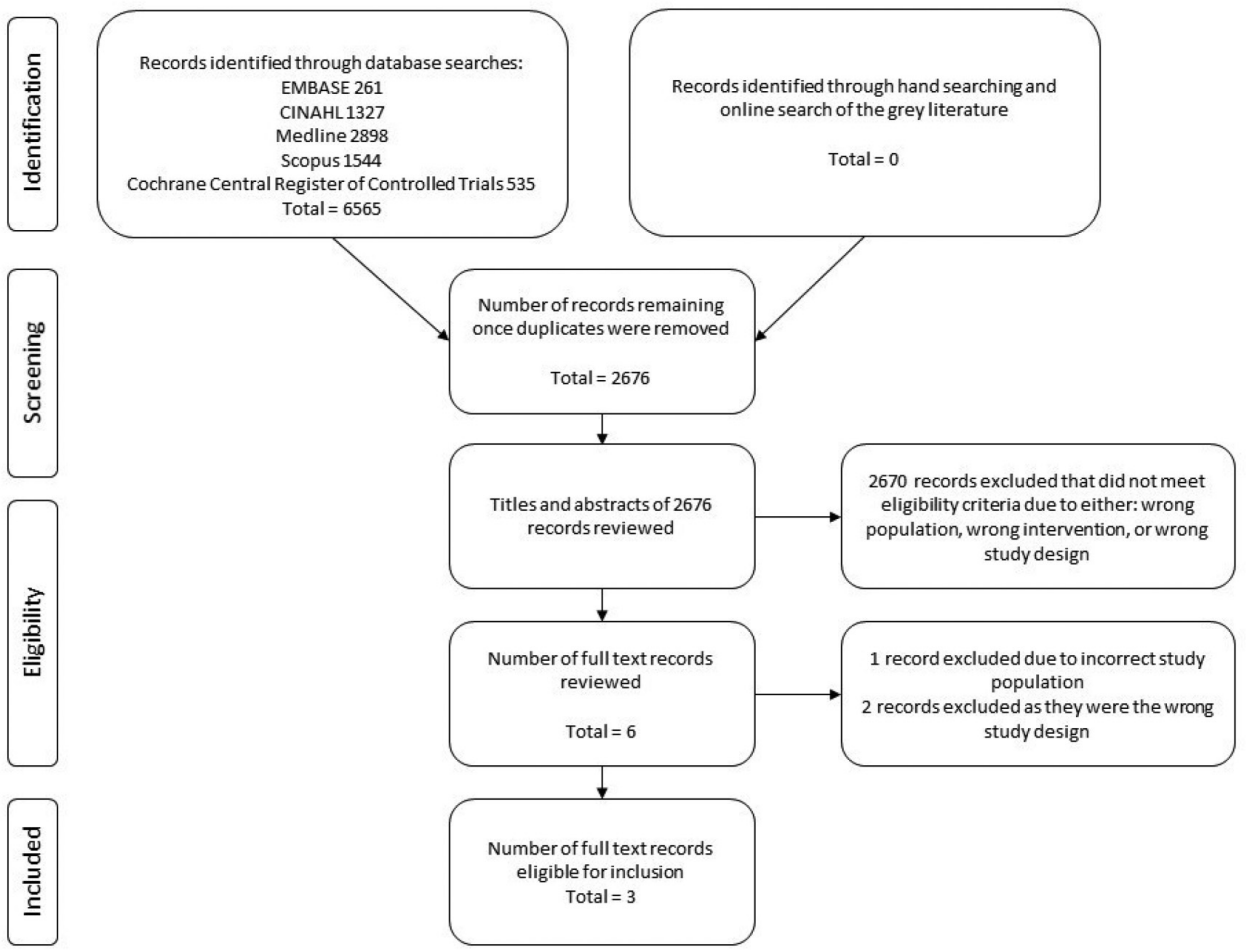
PRISMA flow diagram.

The process of study selection is summarised in Figure 1. No disagreements occurred between reviewers regarding eligibility. One disagreement occurred during the risk of bias assessment relating to a single signalling question on the Cochrane risk-of-bias tool for randomised controlled trials, for the outcome of ‘healing of the lesser tuberosity osteotomy’. This was resolved through discussion.

None of the three published papers provided sufficient information to facilitate a meta-analysis. Corresponding authors were contacted to request access to the required data. Two authors did not respond despite two separate requests being sent. A narrative synthesis was therefore undertaken.

### Characteristics of included studies

Table 2 provides an overview of the characteristics of the three included randomised controlled trials. 230 participants were recruited across the three trials: 60 (26%) had an anatomic total shoulder replacement with a lesser tuberosity osteotomy^12^ and 170 (74%) had a reverse total shoulder replacement without repair of the subscapularis.^17,18^ 204 patients were included in the final analyses; 100 were female and 104 were male. The mean age of participants ranged from 68.3 to 75.1 years.

**Table 2.** Characteristics of included studies.

*Author* *Year of publication*	*Study setting* *Sample size* *Sample population* *Prosthesis used*	*Intervention* *(Early rehabilitation ER)*	*Control/comparator* *(Delayed rehabilitation DR)*	*Outcome measures &* *Data collection time-points*	*Findings*
**Denard and Lädermann 2016** ^12^	**Setting** Orthopaedic hospital setting (full details not given in the paper) **Sample size** 60 enrolled (55 completed 12-month follow-up) **Sample population** Patients undergoing anatomic unconstrained Total shoulder arthroplasty with a lesser tuberosity osteotomy **Prosthesis** Short-stem press-fit humeral component. All polyethylene glenoid components used: 50 pegged components, 10 keeled components. Manufacturer details not given.	**Sling for 4 weeks** **Day 1 post-op:** Passive FF with pulley. Passive ExR to 30 deg. Active movements of the hand, wrist, elbow and scapular retraction also allowed immediately post-op.	**Sling for 4 weeks.** **During the first 4 weeks**, no movement of the shoulder; only active movement of the elbow, wrist and hand, and active scapular retraction allowed.	**ROM, SST, SANE,** **VAS-P, ASES:** Baseline, 4 weeks, 8 weeks, 3 months, 6 months, 12 months post-surgery **Belly Press Test:** 3 months, 6 months, 12 months post-surgery **Healing of the lesser tuberosity osteotomy:** 12 months post-surgery	**ROM**: Between group comparisons at 12 months: FF mean 142° SD 20° ER group, mean 146° SD 20° DR group, *P* = 0.886, ExR mean 62° SD 16° ER group, 57° SD 12° DR group, *P* = 0.209, IR L3 ER group, L1 DR group, *P* = 0.685 **SST**: Between group comparison at 12 months: mean 9.9 SD 2.5 ER group, mean 9.8 SD 2.4 DR group, *P* = 0.376 **SANE**: Between group comparison at 12 months: mean 85.8 SD 20.6 ER group, mean 88.2 SD 12.4 DR group, *P* = 0.940. Intervention group more improved at 8 weeks *P* = 0.012, point estimates not provided **VAS-P**: Between group comparison at 12 months: mean 0.7 SD 0.9 ER group, mean 1.0 SD 1.4 DR group, *P* = 0.535. Between group comparison at 8 weeks *P* = 0.019 showed greater improvement in the intervention group. Point estimates not given. **ASES**: Between group comparison at 12 months: mean 89.0 SD 10.9 ER group, mean 88.9 SD 13.1 DR group, *P* = 0.394. ASES scores were significantly better in the intervention group at 4 weeks (*P* = 0.025) and 8 weeks (*P* = 0.010). Point estimates not given. **Belly press test**: Direct between-group comparison not given - authors state belly press more likely to be negative when the osteotomy was healed *P* < 0.001 **Healing of the lesser tuberosity osteotomy:** No statistically significant difference between the groups at 12 months 81.5% ER group, 96.4% DR group, *P* = 0.101^a^
		**Week 4:** Sling discontinued. Passive ExR allowed as tolerated. FF progressed from active to assist to active motion as tolerated.	**Week 4:** Passive FF with pulley and passive ExR with a stick as tolerated.		
		**Week 8:** Strengthening initiated.	**Week 8:** AAROM progressing to AROM as tolerated. Strengthening also routinely started at 8 weeks.		
		**Week 12:** Activities allowed as tolerated. Lifetime recommendation of no repetitive lifting of over 11.3 Kg (25 lb).	**Week 16**: Activities allowed as tolerated. Lifetime recommendation of no repetitive lifting of over 11.3 Kg (25 lb).		
**Hagen et al.** **2020** *^17^*	**Setting** University Medical Centre USA **Sample size** 107 enrolled (86 completed 12-month follow-up, 65 completed 2-year follow-up) **Sample population** Patients undergoing reverse total shoulder arthroplasty without repair of subscapularis **Prosthesis** Zimmer Biomet Trabecular Metal reverse shoulder system	Early rehabilitation: Sling worn for 6 weeks but can be removed for showering, exercises and other daily activities as needed. Scapular exercises begun immediately.	Delayed rehabilitation: sling immobilisation with no passive or active movement of the shoulder for 6 weeks. Sling worn throughout the day and for sleeping.	**AROM, PROM, ASES:** Baseline, 6 weeks, 3 months, 6 months, 12 months, (2 years) post-surgery **Scapular notching:** 12 months post-surgery	**AROM**: Between group analysis of the change in ROM from pre-op to each timepoint said to show no statistically significant difference- figures not given^a^ **PROM**: Between group analysis of the change in ROM from pre-op to each timepoint said to show no statistically significant difference- figures not given^a^ **ASES**: No between-group difference in mean change from baseline seen at any timepoint except 6 months: ASES pain and composite scores better for the delayed therapy group; pain score mean change from baseline 16.7 SD 11.6 ER group, mean change 26.3 SD 16.3 DR group, *P* = 0.008, composite score mean change from baseline 30.0 SD 18.8 ER group, mean change 40.2 SD 20.1 DR group, *P* = 0.038 **Scapular notching**: Statistical comparison not attempted. (Intervention group - 27 cases of Nerot-Sirveaux class 0, 13 cases of class 1 and 2 cases of class 2. Control group - 20 cases of Nerot-Sirveaux class 0, and 24 cases of class 1)
		**Week 1:** PROM begins at 7-10 days FF, ExR and abd. Pendulum exercises commenced.	**Day 1:** Active scapular retraction/shoulder shrug exercises begun immediately.		
		**Weeks 1-12:** Gradual introduction of AAROM. Whilst progressing with AAROM, the patient begins AROM FF, Abd and ER	**Week 6**: Begin PROM: FF, Abd, ExR. Pendular exercises also begun at 6 weeks. Gradual introduction of AAROM, progressing to AROM during the 6-12-week period.		
		**Weeks 6-12:** Introduction of isometric strengthening **Weeks 12 onwards:** Resisted exercises	**Weeks 6-12**: Patient may begin isometric strengthening when comfortable with AROM. **Week 12 onwards:** Resisted exercises		
**Edwards** **et al.** **2020** *^18^*	**Setting** Orthopaedic surgical hospital/ clinic **Sample size** 63 enrolled (55 completed 12-month follow-up but all 63 retained in the final analysis) **Sample population** Patients undergoing reverse total shoulder arthroplasty without repair of subscapularis. **Prosthesis** All patients had a prosthesis with a medial glenoid and lateralised humerus design. Exactech Equinoxe Reverse Shoulder System	**Weeks 0-2:** Immobilisation	**Weeks 0-2:** Immobilisation	**ASES, VAS-P, GSF, SANE, AQOL-4D, SAS:** Baseline, 3 months, 6 months, 12 months post-surgery **Constant score, ROM, Peak isometric shoulder strength:** 3 months, 6 months, 12 months post-surgery	**ASES, VAS-P, GSF, SANE, AQOL-4D and SAS presented as mean within group change (95% CI) for ER and DR groups and result of analyses of between group difference for improvement from baseline.** NB. No statistically significant between-group differences were seen at any time point. **ASES** 3 months: 39.5 (33.0 to 45.9) ER group, 36.2 (30.0 to 42.3) DR group, p = 0.466. 6 months: 50.3 (42.8 to 57.8) ER group, 46.0 (-6.3 to 14.8) DR group, *P* = 0.428. 12 months: 57.0 (50.4 to 63.7) ER group, 52.5 (46.8 to 58.1) DR group, *P* = 0.306 **VAS-P**: 3 months: -5.0 (-5.9 to -4.2) ER group, -4.4 (-5.1 to -3.7) DR group, *P* = 0.282 6 months, -5.6 (-6.5 to – 4.8) ER group, -4.7 (-5.7 to -3.8) DR group, *P* = 0.161 12 months: -6.2 (-7.0 to -5.5) ER group, -5.4 (-6.1 to -4.7), DR group, *P* = 0.080 **GSF**: 3 months: 3.8 (3.0 to 4.6) ER group, 3.2 (2.3 to 4.2) DR group, *P* = 0.384 6 months: 4.9 (4.1 to 5.7) ER group, 3.8 (2.9 to 4.7) DR group, *P* = 0.075 12 months: 5.5 (4.4 to 6.5) ER group, 4.8 (4.0 to 5.6), DR group, *P* = 0.362 **SANE**: 3 months: 36.7 (26.0 to 47.4) ER group, 31.4 (22.9 to 39.9) DR group, *P* = 0.448 6 months: 49.4 (40.7 to 58.1) ER group, 40.8 (32.8 to 48.7) DR group, *P* = 0.158 12 months: 55.6 (47.0 to 64.2) ER group, 47.1 (39.5 to 54.7) DR group, *P* = 0.128 **AQOL-4D**: 3 months: 10.0 (3.0 to 17.0) ER group, 9.6 (3.1 to 16.1) DR group, *P* = 0.942 6 months: 13.3 (3.5 to 23.2) ER group, 16.1 (5.0 to 27.3) DR group, *P* = 0.886 12 months: 12.9 (4.9 to 20.9) ER group, 14.9 (6.2 to 23.6) DR group, *P* = 0.653 **SAS**: 12 months: 5.1 (2.3 to 7.9) ER group, 6.3 (2.7 to 9.8) DR group, *P* = 0.605 **Constant score**: No significant group differences or interaction (*P* > 0.05) at any time point **ROM:** No significant between group differences or interaction effects were observed in FF, Abd, ExR or IR (*P* > 0.05). Post hoc t-tests revealed significantly better (*P* = 0.019) FF at 3 months post-surgery for the ER group. **Peak Isometric shoulder strength:** No significant between group differences or interaction effects were observed in FF, Abd, ExR and IR peak isometric strength at 3, 6, or 12 months post-surgery (*P* > 0.05). A significant between group difference for improvement existed for FF strength at the three to six-month interval (mean difference 0.8; 95% CI: -1.5 to -0.4; *P* = 0.038) favouring the DR group.
		**Weeks 2-6:** Continue with sling use. Commencement of PROM and AAROM. Submaximal isometric contraction of Deltoid (ant/mid/post).	**Weeks 2-6:** Continued use of sling and commencement of PROM - FF to 90 degrees, pendular exercises and self-assisted ExR.		
		**Weeks 6-12:** Discontinue use of sling. Progression of AAROM to AROM. Active ExR strengthening using yellow Theraband/0.5-1 kg dumbell.	**Weeks 6-12:** Discontinue use of sling. Progression of PROM to AAROM: Pulley assisted elevation and assisted upright wall slides.		
		**Weeks 12-20**: Active shoulder strengthening- progressive resisted exercises using Theraband or dumbell.	**Weeks 12-20:** Activity as tolerated		

Abbreviations: ROM Range of movement, AROM Active range of movement, AAROM Active assisted range of movement, PROM Passive range of movement, SST Simple shoulder test, SANE Single assessment numeric evaluation, ASES American shoulder and elbow surgeon's score, VAS-P Visual analogue pain scale, GSF Global shoulder function, AQOL-4D Four-dimension version of the assessment of quality of life, SAS Shoulder activity scale, FF Forward flexion, ExR External rotation, IR Internal rotation, Abd Abduction, ER Early rehabilitation, DR Delayed rehabilitation, SD Standard deviation.

a Only findings from selected time points were reported in the published paper.

The follow-up time points varied (Table 2). Early post-operative outcomes (less than three months) were recorded in two randomised controlled trials;^12,17^ one randomised controlled trial recorded the first post-operative measurements at three months.^18^ Each included trial recorded outcomes at 12 months after the surgery^12,17,18^ and one recorded final outcomes at two years.^17^

### Rehabilitation protocols

The definitions of ‘early’ and ‘delayed’ rehabilitation varied across the three randomised controlled trials. Table 3 summarises the rehabilitation protocols employed, demonstrating the differences within and between the included trials.

**Table 3.** Summary of early and delayed rehabilitation protocols for all included RCTs.

Type of activity	Denard and Lädermann 2016		Hagen et al. 2020		Edwards et al. 2020	
	Early Rehabilitation	Delayed Rehabilitation	Early Rehabilitation	Delayed Rehabilitation	Early Rehabilitation	Delayed Rehabilitation
Post-operative shoulder immobilisation (sling use)	4 weeks: Sling removed for exercises, then discontinued at 4 weeks	4 weeks: Strict shoulder immobilisation for 4 weeks then sling discontinued	6 weeks: Sling can be removed as required for personal care, exercises and ADL, then discontinued at 6 weeks	6 weeks: Strict shoulder immobilisation for 6 weeks then sling discontinued	6 weeks: Strict shoulder immobilisation for 2 weeks. Sling removed for exercises thereafter. Sling use discontinued at 6 weeks	6 weeks: Strict shoulder immobilisation for 2 weeks. Sling removed for exercises thereafter. Sling use discontinued at 6 weeks.
Passive movement	Day 1: Passive FF and ExR to 30°	4 weeks: Passive FF and ExR as tolerated	Day 7-10: Passive FF, ExR and Abd	6 weeks: Passive FF, ExR and Abd	2 weeks: Commence passive movement as tolerated	2 weeks: Passive FF to 90° only
Active-assisted movement	Not specified	8 weeks	1-12 weeks: Gradual introduction	6 weeks: Gradual introduction	2 weeks	6-12 weeks: Progression of passive to active assisted
Active movement	4 weeks: Active FF allowed as tolerated	8 weeks: Gradual progression of active assisted to active	1-12 weeks: Gradual progression of active assisted to active	6-12 weeks: Gradual progression of active assisted to active	6 weeks: Progression of active assisted movement to active	Not specified: 12 weeks activity as tolerated
Isometric shoulder strengthening	8 weeks: Strengthening initiated – type of strengthening not specified	8 weeks: Strengthening initiated – type of strengthening not specified	6-12 weeks	6-12 weeks: May begin isometric strengthening when comfortable with active ROM	2 weeks: Submaximal isometric deltoid contractions	Not specified: 12 weeks activity as tolerated
Resisted shoulder strengthening	8 weeks: Strengthening initiated – type of strengthening not specified	8 weeks: Strengthening initiated – type of strengthening not specified	12 weeks	12 weeks	6 weeks: Active ExR strengthening 12 weeks: Progressive resisted strengthening	Not specified: 12 weeks activity as tolerated

Abbreviations: ADL Activities of daily living, FF Forward flexion, ExR External rotation, Abd Abduction, ROM Range of movement.

### Risk of bias

**Figure 2.**
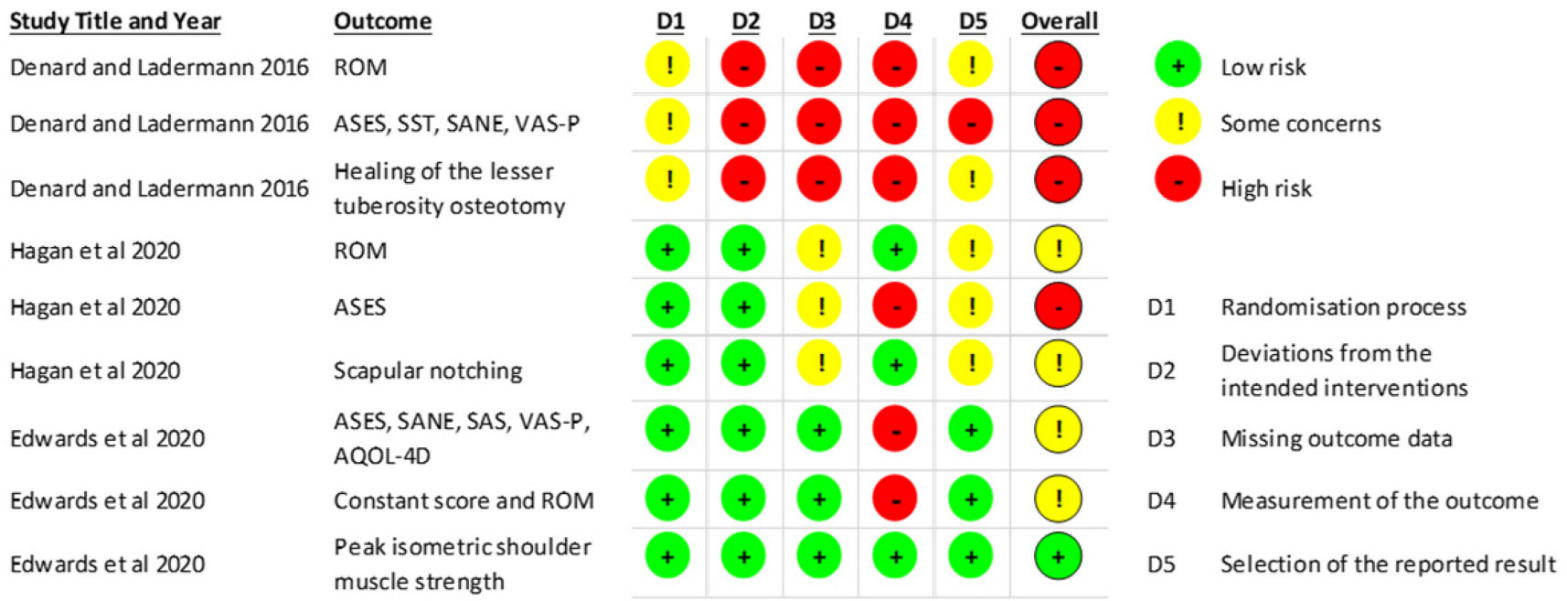
Cochrane risk of bias assessment overview

**Abbreviations:** ROM range of movement, SST simple shoulder test, SANE single assessment numeric evaluation, ASES american shoulder and elbow surgeon's score, VAS-P visual analogue pain scale, GSF global shoulder function, AQOL-4D four-dimension version of the assessment of quality of life, SAS shoulder activity scale.

Figure 2 describes the overall risk of bias assessment. A detailed overview of the domain level assessment of risk of bias can be found in the supplementary file 2. For full details of the GRADE assessment see supplementary file 3.

### Outcome measures

The heterogeneity in the choice of outcome measures across the three included trials is demonstrated in Table 2.

### Outcomes

#### Pain

The two randomised controlled trials that measured pain as an outcome used visual analogue scales. Edwards et al.^18^ used a zero to ten scale; Denard and Lädermann^12^ did not provide such detail. No statistically significant differences between groups were found at three, six or twelve months in either study. Denard and Lädermann however found a statistically significant difference between groups at eight weeks favouring the early rehabilitation group (*P* = 0.019, point estimates not provided).^12^ Such early outcomes were not recorded by Edwards et al.^18^

Grading of evidence: There is very low-quality evidence that early rehabilitation improves pain compared with delayed rehabilitation in the early post-operative period.

### Composite measures of shoulder pain and function

Each included randomised controlled trial used the American Shoulder and Elbow Surgeon's Score (ASES) as a composite measure of shoulder pain and function. Denard and Lädermann^12^ reported statistically significantly superior composite ASES scores in the early rehabilitation group at four weeks and eight weeks following surgery (*P* = 0.025 and *P* = 0.010 respectively). Point estimates were not provided therefore it is not known whether the between-group difference exceeded the minimum clinically important difference. In contrast, Hagen et al.^17^ reported no statistically significant difference between groups in change in American Shoulder and Elbow Surgeon's Score from baseline to six weeks (*P* value and point estimates not reported). At six months post-surgery, Edwards et al.^18^ found no statistically significant difference between the groups; Hagen et al.^17^ however reported a difference in change in composite scores from baseline favouring the delayed rehabilitation group (mean improvement 40.2 + /− SD 20.1 in the delayed therapy group, 30.0 + /− SD 18.8 in the early therapy group, *P* = 0.038). This difference is smaller than the minimum clinically important difference of 21 points.^19^

Grading of evidence: There is very low-quality conflicting evidence evaluating the effect of early and delayed rehabilitation on composite outcomes of shoulder pain and function.

### Shoulder function

Both Denard and Lädermann^12^ and Edwards et al.^18^ used the Single Assessment Numeric Evaluation (SANE) to assess shoulder function, but only Edwards et al.^18^ provided point estimates for SANE scores. The only statistically significant difference between the groups was found by Denard and Lädermann at eight weeks, in favour of the early rehabilitation group (*P* = 0.012, point estimates not provided).^12^

Denard and Lädermann^12^ reported no statistically significant difference in Simple Shoulder Test (SST) scores between the groups at 12 months post-surgery (Mean SST score 9.9 + /− SD 2.5 early rehabilitation group versus mean SST score 9.8 + /− SD 2.4 delayed rehabilitation group, *P* = 0.376). Similarly, Edwards et al.^18^ reported no statistically significant between-group differences in change from baseline for the Shoulder Activity Scale, Constant-Murley, or the Global Shoulder Function scores at any of the time points recorded.

Grading of evidence: There is very low-quality conflicting evidence evaluating the effect of early and delayed rehabilitation on shoulder function.

### Health-related quality of life

Only Edwards et al.^18^ specifically measured health-related quality of life, using the four-dimension version of the Assessment of Quality of Life (AQOL-4D). No statistically significant differences in change from baseline were seen between the early and delayed groups at three, six or 12 months (mean change from baseline 65.7 + /− SD 19.3 early group, 71.6 + /− SD 20.4 delayed group *P* = 0.942; 69.5 ± SD 24.3 early group, 78.3 ± SD 14.8 delayed group, *P* = 0.886; and 68.6 ± SD 18.0 early group, 77.7 ± SD 17.7 delayed group, *P* = 0.653 respectively).

Grading of evidence: There is very low-quality evidence of no difference in improvement in health-related quality of life up to 12 months following total shoulder replacement with early or delayed rehabilitation.

### Range of movement

Each of the three randomised controlled trials measured active range of movement in all participants. Denard and Lädermann^12^ used a goniometer to measure shoulder range of movement into forward flexion and external rotation with the patient's arm at their side; internal rotation was estimated using the highest spinal level reached. Edwards et al.^18^ reported use of a goniometer to measure forward flexion, abduction and external rotation with the patient in a supine position, and assessed internal rotation by the highest spinal level reached. Hagen et al.^17^ recorded both active and passive range of movement into forward flexion, abduction, external rotation and cross-body adduction, but the method of measurement was not stated.

Denard and Lädermann^12^ found no statistically significant difference between groups at 12 months for forward flexion, external rotation and internal rotation (Mean forward flexion 142° ± SD 20° early rehabilitation group, mean 146° ± SD 20° delayed group, *P* = 0.886, mean external rotation 62° ± SD 16 early rehabilitation group, mean 57° ± SD 12 delayed group, *P* = 0.209, and mean internal rotation L3 early rehabilitation group, L1 delayed group, *P* = 0.685 respectively). Hagen et al.^17^ did not present descriptive data, however reported no statistically significant differences in change in range of movement from baseline between groups at any time point. Edwards et al.^18^ reported that within-group time effects were observed for both groups from three to six and 12 months post-surgery, and post-hoc t-tests revealed significantly better forward flexion measurements in the early rehabilitation group at three months post-surgery (additional data provided by the author: mean 140.50, 95% CI 135.101–145.899 early rehabilitation group, mean 131.240, 95% CI 125.734–136.746 delayed group, *P* = 0.019).

Grading of evidence: There is very low-quality conflicting evidence evaluating the effect of early and delayed rehabilitation on shoulder range of movement up to 12 months post-surgery.

### Peak isometric shoulder strength

Edwards et al.^18^ measured peak isometric shoulder muscle strength in kilograms using a digital hand-held dynamometer. The authors state that no significant between-group differences or interaction effects were observed for forward flexion, abduction, external rotation or internal rotation peak isometric strength at three, six, or 12 months post-surgery (*P* > 0.05, point estimates not provided in the published paper).

Grading of evidence: There is low-quality evidence suggesting that early and delayed rehabilitation result in no significant difference in peak isometric shoulder strength at three, six, and 12 months post-surgery.

### Healing of the lesser tuberosity osteotomy

Denard and Lädermann^12^ reported no statistically significant difference (*P* = 0.101) in healing rates of the lesser tuberosity visible on plain X-Ray in the delayed (96.4%) versus early rehabilitation group (81.5%) at 12 months.

Grading of evidence: There is very low-quality evidence demonstrating no difference in healing of the lesser tuberosity osteotomy at 12 months following early or delayed rehabilitation.

## Discussion

This systematic review was undertaken to assess the effectiveness of early versus delayed post-operative rehabilitation following total shoulder replacement. There was substantial heterogeneity in the interventions employed and insufficient data to allow statistical pooling of results via meta-analysis. The available evidence, which is of very low to low methodological quality, suggests no difference in patient-reported or clinician-reported outcomes at 12 months post-surgery.^12,17,18^ There is however some limited, very low-quality evidence of improved pain and functional outcomes scores^12^ in the first eight weeks post-surgery in those undergoing early rehabilitation following anatomic total shoulder replacement.

Across the three included randomised controlled trials, the definitions of early rehabilitation varied considerably, with differences noted in sling use and the timeframes for commencement of active shoulder movement and strengthening.^12,17,18^ Furthermore, what was termed ‘early’ rehabilitation in the included randomised controlled trials (e.g. beginning passive movement on the first post-operative day) would, in some settings, be considered standard practice.^9^

Variability was seen in the choice of patient reported outcome measures selected, with just the American Shoulder and Elbow Surgeon's Score used consistently across the three included randomised controlled trials. Only Edwards et al.^18^ opted to measure health-related quality of life in addition to shoulder specific functional outcomes. Yet sling immobilisation, shoulder pain, and limited shoulder range of movement may all have a wider impact on the patient than joint-specific measures can detect.^20^ Future research should consider a more holistic approach and include outcome measures that capture the impact on social function and health-related quality of life that shoulder pain and restriction can create.

In this review, randomised controlled trials that included participants undergoing both anatomic and reverse total shoulder replacement were included despite recommendations by some that the rehabilitation programs for the two procedures should differ.^6,8^ A recent review of publicly available rehabilitation protocols published by UK NHS Trusts reported that few institutions differentiate rehabilitation protocols based on prosthesis type. For those that do, there is little difference between the two approaches,^9^ hence the decision to include what some may consider to be two clinically distinct patient groups.

One reason commonly proposed for a more conservative postoperative approach following anatomic total shoulder replacement is to protect the subscapularis repair or lesser tuberosity osteotomy.^8^ A functioning subscapularis is thought to be important to maintain the balance of the rotator cuff.^21^ A structurally, or functionally, deficient rotator cuff can increase the shear forces and edge-loading of an anatomic shoulder replacement, increasing the likelihood of early glenoid component loosening and eventual revision surgery.^22^ For this reason a more conservative rehabilitation approach, with a period of strict shoulder immobilisation, is often recommended.^8^ Denard and Lädermann^12^ reported lower rates of lesser tuberosity osteotomy healing in the early rehabilitation group but this difference did not reach statistical significance. Despite this, healing rates need to be carefully monitored in future adequately powered randomised controlled trials.

Finally, this review found that there is limited, very low-quality evidence from one RCT that early rehabilitation may result in better functional outcome scores at four weeks and eight weeks following anatomic total shoulder replacement compared to delayed rehabilitation. Point estimates were not provided in the published paper therefore it is not known whether the between-group difference exceeded the minimum clinically important differences for the chosen outcome measures. Nonetheless, recent research has demonstrated that improved post-operative function following total shoulder replacement is associated with higher patient satisfaction.^23,24^ Improved functional independence also results in reduced patient frustration as reliance on others for self-care is reduced.^20^ An earlier return to function may therefore be an important outcome for patients even if the advantages of early rehabilitation are no longer visible by three-month post-surgery. The impact of early rehabilitation during the first eight weeks following surgery therefore warrants further exploration in future high-quality randomised controlled trials. Understanding patients’ perspectives on early post-surgical recovery through further qualitative research would also be useful.

The inclusion of three eligible randomised controlled trials in this review highlights the paucity of evidence available to guide clinical practice and underscores the need for further research into this area. Furthermore, the heterogeneity of the interventions included in this review illustrates the clinical uncertainty surrounding rehabilitation after total shoulder replacement. This review has also demonstrated the variability in the use of shoulder-related outcome measures which precludes statistical pooling of results. Although this might be problematic, standardisation of outcome measures is not always desirable, and outcome selection should be directed by what matters to patients and their communities.

The strengths of this systematic review include pre-publication of the study protocol; adherence to methodological guidance, including an in-depth assessment of the quality of available evidence; and the inclusion of two independent reviewers at every stage of the review. This systematic review was however limited to randomised controlled trials published in English, which limits the scope. There was also insufficient data available to allow a meta-analysis and only a small number of trials eligible for inclusion. The definitions of early and delayed rehabilitation employed in each study varied, as did the outcome measures chosen.

Future research should focus on the uncertainties surrounding the optimal time point at which to begin mobilisation of the shoulder following surgery and how long the post-operative sling should be worn. Understanding patients’ perspectives on early post-surgical recovery through further qualitative research would also be useful in helping to prioritise patient-important outcomes in the early post-operative period.

### Clinical messages

- This systematic review found no differences in patient-reported or clinician-reported outcomes at 12 months post-surgery between early rehabilitation and delayed rehabilitation following total shoulder replacement.
- There is some limited, very low-quality evidence of improved pain and functional outcomes in the early post-operative phase with early rehabilitation following anatomic total shoulder replacement.

## Supplemental Material

sj-docx-1-cre-10.1177_02692155211044137 - Supplemental material for Effectiveness of early versus delayed rehabilitation following total shoulder replacement: A systematic review Click here for additional data file. Supplemental material, sj-docx-1-cre-10.1177_02692155211044137 for Effectiveness of early versus delayed rehabilitation following total shoulder replacement: A systematic review by Maria Moffatt, Gareth Whelan, Peter Gill, Bruno Mazuquin, Peter Edwards, Chris Peach, Ronnie Davies, Marie Morgan and Chris Littlewood in Clinical Rehabilitation

sj-docx-2-cre-10.1177_02692155211044137 - Supplemental material for Effectiveness of early versus delayed rehabilitation following total shoulder replacement: A systematic review Click here for additional data file. Supplemental material, sj-docx-2-cre-10.1177_02692155211044137 for Effectiveness of early versus delayed rehabilitation following total shoulder replacement: A systematic review by Maria Moffatt, Gareth Whelan, Peter Gill, Bruno Mazuquin, Peter Edwards, Chris Peach, Ronnie Davies, Marie Morgan and Chris Littlewood in Clinical Rehabilitation

sj-docx-3-cre-10.1177_02692155211044137 - Supplemental material for Effectiveness of early versus delayed rehabilitation following total shoulder replacement: A systematic review Click here for additional data file. Supplemental material, sj-docx-3-cre-10.1177_02692155211044137 for Effectiveness of early versus delayed rehabilitation following total shoulder replacement: A systematic review by Maria Moffatt, Gareth Whelan, Peter Gill, Bruno Mazuquin, Peter Edwards, Chris Peach, Ronnie Davies, Marie Morgan and Chris Littlewood in Clinical Rehabilitation
