# Supplementary material for: Effectiveness of early versus delayed rehabilitation following total shoulder replacement: A systematic review
Source: Clin Rehabil. 2021 Nov 1;36(2):190–203. doi: 10.1177/02692155211044137 (PMC8807994; doi:10.1177/02692155211044137)
Supplement: sj-docx-1-cre-10.1177_02692155211044137 - Supplemental material for Effectiveness of early versus delayed rehabilitation following total shoulder replacement: A systematic review [file sj-docx-1-cre-10.1177_02692155211044137.docx]

**Search strategies for online databases:**

**MEDLINE:**

**Search strings used**

Shoulder arthroplast * OR shoulder replacement* OR Glenohumeral arthroplasty* OR glenohumeral replacement*

AND

Exercise* OR Rehab* OR Physiother* OR Physical ther* OR Telerehabilitation OR e-rehabilitation or m-rehabilitation

**MESH Terms also searched (All expanded/exploded)**

Shoulder

Shoulder joint

Shoulder, arthroplasty, replacement

Physical therapy

Exercise

**CINAHL:**

**Search strings used**

Shoulder arthroplast * OR shoulder replacement* OR Glenohumeral arthroplasty* OR glenohumeral replacement*

AND

Exercise* OR Rehab* OR Physiother* OR Physical ther* OR Telerehabilitation OR e-rehabilitation or m-rehabilitation

**CINAHL subject headings also searched**

Shoulder

Arthroplasty, replacement, shoulder

Arthroplasty, reverse, total, shoulder

Physical therapy (exploded)

Telerehabilitation (Exploded)

Rehabilitation (Exploded)

Exercise (Exploded)

**SCOPUS:**

**Search strings used**

Shoulder arthroplast * OR shoulder replacement* OR Glenohumeral arthroplasty* OR glenohumeral replacement*

AND

Exercise* OR Rehab* OR Physiother* OR Physical ther* OR Telerehabilitation OR e-rehabilitation or m-rehabilitation

**EMBASE**

**Search strings used**

Shoulder arthroplast * OR shoulder replacement* OR Glenohumeral arthroplasty* OR glenohumeral replacement*

AND

Exercise* OR Rehab* OR Physiother* OR Physical ther* OR Telerehabilitation OR e-rehabilitation or m-rehabilitation

AND

Randomised controlled trial OR Randomized controlled trial OR Randomised trial OR Randomized trial OR Controlled trial OR Clinical trial OR Trial OR Pragmatic trial

**Cochrane CENTRAL Trials database:**

**Search strings used**

Shoulder arthroplast * OR shoulder replacement* OR Glenohumeral arthroplasty* OR glenohumeral replacement*

AND

Exercise* OR Rehab* OR Physiother* OR Physical ther* OR Telerehabilitation OR e-rehabilitation or m-rehabilitation

**All searches limited to English and journal articles. Title, abstract and keywords searched.**
