# Supplementary material for: Effectiveness of early versus delayed rehabilitation following total shoulder replacement: A systematic review
Source: Clin Rehabil. 2021 Nov 1;36(2):190–203. doi: 10.1177/02692155211044137 (PMC8807994; doi:10.1177/02692155211044137)
Supplement: sj-docx-2-cre-10.1177_02692155211044137 - Supplemental material for Effectiveness of early versus delayed rehabilitation following total shoulder replacement: A systematic review [file sj-docx-2-cre-10.1177_02692155211044137.docx]

**Supplementary file 1.** Cochrane risk of bias assessment: all domains for all outcomes

| Study | Outcome | Domain 1:  RoB from randomisation process | Domain 2: RoB due to deviation from intended intervention | Domain 3: RoB due to missing outcome data | Domain 4: RoB due to measurement of the outcome | Domain 5: RoB in selection of reported results |
| --- | --- | --- | --- | --- | --- | --- |
| Denard and Lädermann 2016 | ROM |  |  |  |  |  |
|  | SST |  |  |  |  |  |
|  | SANE |  |  |  |  |  |
|  | ASES |  |  |  |  |  |
|  | VAS P |  |  |  |  |  |
|  | Belly Press Test |  |  |  |  |  |
|  | Osteotomy Healing |  |  |  |  |  |
|  |  |  |  |  |  |  |
| Hagen et al 2020 | AROM |  |  |  |  |  |
|  | PROM |  |  |  |  |  |
|  | ASES |  |  |  |  |  |
|  | Scapular notching |  |  |  |  |  |
|  |  |  |  |  |  |  |
| Edwards et al 2020 | ASES |  |  |  |  |  |
|  | VASP |  |  |  |  |  |
|  | GSF |  |  |  |  |  |
|  | SANE |  |  |  |  |  |
|  | AQOL-4D |  |  |  |  |  |
|  | SAS |  |  |  |  |  |
|  | Constant Score |  |  |  |  |  |
|  | AROM |  |  |  |  |  |
|  | Isometric Strength |  |  |  |  |  |

High risk of bias

Some concerns

Low risk of bias

**Supplementary file 1. Abbreviations:** RoB Risk of bias, ROM Range of movement, AROM Active range of movement, PROM Passive range of movement, SST Simple shoulder test, SANE Single assessment numeric evaluation, ASES American shoulder and elbow surgeon’s score, VAS-P Visual analogue pain scale, GSF Global shoulder function, AQOL-4D Four-dimension version of the assessment of quality of life, SAS Shoulder activity scale
