# Supplementary material for: Effectiveness of early versus delayed rehabilitation following total shoulder replacement: A systematic review
Source: Clin Rehabil. 2021 Nov 1;36(2):190–203. doi: 10.1177/02692155211044137 (PMC8807994; doi:10.1177/02692155211044137)
Supplement: sj-docx-3-cre-10.1177_02692155211044137 - Supplemental material for Effectiveness of early versus delayed rehabilitation following total shoulder replacement: A systematic review [file sj-docx-3-cre-10.1177_02692155211044137.docx]

**Supplementary file 2.** GRADE Assessment: early rehabilitation compared to delayed rehabilitation for individuals following anatomic or reverse total shoulder arthroplasty

| **Certainty assessment** | | | | | | | **Certainty** |
| --- | --- | --- | --- | --- | --- | --- | --- |
| **№ of studies** | **Study design** | **Risk of bias** | **Inconsistency** | **Indirectness** | **Imprecision** | **Other consider-ations** |  |
| **Pain (Denard 2016, Edwards 2020) (assessed with: Visual analogue scale)** | | | | | | | |
| 2 | randomised trials | very serious ^a^ | not serious | not serious | serious ^b^ | none | ⨁◯◯◯ VERY LOW |
| **Shoulder function (Denard 2016, Hagen 2020, Edwards 2020) (assessed with: ASES (Denard 2016, Hagen 2020, Edwards 2020), SANE (Denard 2016, Edwards 2020), SST (Denard 2016), GSF (Edwards 2020), SAS (Edwards 2020), Constant Score (Edwards 2020))** | | | | | | | |
| 3 | randomised trials | very serious ^c^ | not serious | not serious | serious ^d^ | none | ⨁◯◯◯ VERY LOW |
| **Range of shoulder movement (Denard 2016, Hagen 2020, Edwards 2020) (assessed with: Goniometer used by Denard 2016 and Edwards 2020 for forward flexion, abduction and external rotation. Method of measurement not specified by Hagen 2020)** | | | | | | | |
| 3 | randomised trials | very serious ^e^ | not serious | not serious | serious ^d^ | none | ⨁◯◯◯ VERY LOW |
| **Health related quality of life (Edwards 2020) (assessed with: Four-dimension version of the assessment of quality of life (AQOL-4D))** | | | | | | | |
| 1 | randomised trials | serious ^f^ | serious ^g^ | not serious | serious ^b,g^ | none | ⨁◯◯◯ VERY LOW |
| **Peak Isometric Shoulder Strength (Edwards 2020) (assessed with: Hand-held digital dynamometer)** | | | | | | | |
| 1 | randomised trials | not serious | serious ^g^ | not serious | serious ^g^ | none | ⨁⨁◯◯ LOW |
| **Healing of the lesser tuberosity osteotomy (Denard 2016) (follow up: mean 12 months; assessed with: Radiological assessment via true glenohumeral anteroposterior and axillary radiographs (Denard 2016))** | | | | | | | |
| 1 | randomised trials | very serious ^h^ | serious ^g^ | not serious | serious ^g^ | none | ⨁◯◯◯ VERY LOW |
| **Scapular notching (Hagen 2020) (follow up: mean 12 months; assessed with: Nerot-Sirveaux classification)** | | | | | | | |
| 1 | randomised trials | not serious | serious ^g^ | not serious | serious ^g^ | none | ⨁⨁◯◯ LOW |

a. ITT analysis not specified and unclear allocation concealment for one study. Lack of adequate blinding of participants and assessors having a possible effect on the outcome in both studies.

b. Small sample sizes for the studies measuring this outcome – total sample for both studies <300 participants

c. Unclear allocation concealment in one study. Selective outcome reporting in one study. Failure to specify ITT analysis in one study. Unclear blinding of participants and outcome assessors in two studies.

d. Small sample sizes for included studies - total sample for this review <300 participants

e. Unclear allocation concealment in one study. Failure to specify ITT analysis in one study. Unclear blinding of participants and outcome assessors in two studies. Method of measurement not specified in one study.

f. Unclear or lack of blinding of assessors.

g. Only one study available that measured this outcome with <300 participants, therefore downgrade for inconsistency and imprecision

h. Unclear allocation concealment. Unclear blinding of participants and assessors. ITT analysis not specified.
